# Influential factors shaping consumers’ green packaging purchase intentions

**DOI:** 10.3389/fpsyg.2025.1730088

**Published:** 2026-01-06

**Authors:** Juan Wang, Guo Li, Ding-Bang Luh

**Affiliations:** School of Art and Design, Guangdong University of Technology, Guangzhou, China

**Keywords:** environmental concern, Greater Bay Area, green packaging, theory of planned behavior, willingness to pay a premium

## Abstract

**Introduction:**

Rapid consumption upgrading and industrial transformation are straining the ecological capacity of China’s Guangdong–Hong Kong–Macao Greater Bay Area, where green packaging is viewed as a crucial means of easing this burden. The conventional theory of planned behavior is limited in explaining green consumption. This study examined green packaging purchasing behavior in the Greater Bay Area by augmenting the conventional theory of planned behavior model to incorporate environmental concern and the willingness to pay a premium.

**Methods:**

The extended model was evaluated using survey data from 370 consumers, employing covariance-based structural equation modeling analysis and mediation effect testing in SPSS 26 and AMOS 28. The results indicate that attitude, perceived behavioral control, and willingness to pay a premium significantly predict consumers’ behavioral intention for green packaging.

**Results:**

Environmental concern plays a pivotal role, not only by amplifying attitudes, perceived behavioral control, and willingness to pay a premium, but also by indirectly influencing behavioral intention. However, subjective norm did not exert a significant effect on behavioral intention. The extended model demonstrated a notable improvement in explanatory power, with R2 rising from 0.710 to 0.859 for behavioral intention and from 0.870 to 0.912 for actual purchase behavior relative to the conventional model.

**Discussion:**

This incorporates value-based antecedsents lacking in the conventional model and elucidates performance improvements. These findings underscore the theoretical significance of incorporating environmental concern and the willingness to pay a premium into the traditional behavioral model, demonstrating that such enhancements markedly improve the forecasting of green purchase behavior.

## 1 Introduction

Climate change, resource depletion, and ecosystem degradation have emerged as significant global challenges, driven by the escalation of environmental issues. In this context, sustainable development has emerged as a matter that consumers and organizations must address, thereby fostering the emergence of green consumerism (Okbagaber, 2023). Emphasis on ecological consumption and consumer protection of the natural environment has also influenced consumers’ purchasing preferences (Chen et al., 2022). Business owners and researchers have begun to focus on green packaging to encourage consumers to adopt environment-friendly practices, as product packaging significantly influences their purchasing behavior (Deng and Yang, 2024). The term “green packaging” typically denotes packaging methods that prioritize reducing resource consumption and environmental pollution throughout the life cycle (design, production, use, and disposal). Its fundamental characteristics include the utilization of biodegradable materials, optimized packaging design, and recycling technology. Presently, researchers have conducted studies on the factors that influence the propensity to purchase green products and engage in green behaviors. A study examining the impact of eco-friendly packaging on consumer purchasing behavior found a significant positive correlation between green packaging and consumer purchasing decisions (Igbomor, 2024). However, research conclusions remain inconsistent. Reviews indicate that the determinants of green purchasing behavior are complex and diverse. The direction and intensity of influence exerted by environmental concerns, economic factors, or consumer beliefs vary considerably across different studies (Yusoff et al., 2023). Simultaneously, research also suggests that even when consumers possess high environmental awareness or positive attitudes toward sustainability, this does not necessarily translate into actual purchasing behavior, demonstrating a classic “value–action” gap (Park and Lin, 2020).

Ajzen’s Theory of Planned Behavior (TPB) is renowned for its capacity to systematically elucidate the psychological mechanisms of consumer behavior (Ajzen, 1991). The TPB is a theoretical framework that elucidates complex consumer behavior by utilizing three central variables—attitude (ATT), subjective norms (SN), and perceived behavioral control (PBC)—to explain an individual’s behavioral intentions (BIs). Nonetheless, the theoretical framework of the TPB has gradually exposed its shortcomings in light of the growing importance of sustainable consumption. For instance, establishing a comprehensive analytical framework for researching green purchasing behavior is challenging because of its inability to effectively convey the influence of external (e.g., environmental concerns; ECs) and market factors (e.g., willingness to pay a premium; WPP) on consumer behavior. Environmental concern is a direct predictor of specific environmental behaviors of consumers, which are predicted by their attitudes toward specific behaviors (Ajzen and Fishbein, 1977). Institutional organizations must comprehend consumers’ propensity to pay a premium for green products, as the price is one of the most significant factors influencing consumer decision-making.

Developed markets in Europe and the United States have been the subject of a significant number of regional studies, whereas studies on developing countries and economies are relatively scarce. As China’s most dynamic world-class urban agglomeration and international science and technology innovation center, the Guangdong-Hong Kong-Macao Greater Bay Area (GBA) offers a unique environment for exploring green packaging purchasing behavior with its well-developed green policy system and high consumption capacity (Zhou et al., 2020). On the one hand, the government has been continuously promoting the carbon peaking and carbon neutrality strategies, which has enhanced the public’s concern for the environment. On the other hand, the relatively high per capita income within GBA enables consumers to have the economic capacity to pay a premium for environmental protection. The investigation of green consumption behavior in this region not only enriches global sustainable development research but also offers theoretical references and practical advice for other emerging economies.

In this study, we employed an extended TPB model to conduct a systematic examination of the driving mechanisms of consumer behavior in the GBA of China, with a focus on the purchase intention of green packaging products. We empirically analyze consumer categories in the GBA using structural equation modeling (SEM) and introduce two new variables: EC and WPP. The objective of this study is to confirm the impact of exogenous variables on green consumer behavior; identify the distinctive characteristics of consumer behavior in the GBA; and establish a scientific foundation for the government, enterprises, and society to develop more practical strategies and policies.

## 2 Literature review and hypothesis development

### 2.1 The theory of planned behavior

The TPB paradigm, as proposed by Ajzen (1991), posits that final decision-making behavior is directly influenced by behavioral ATTs, SN, and the PBC of decisions, which are the basis of individuals’ decision intentions. As defined by Fishbein and Ajzen (1977), human ATTs have a substantial impact on decision intentions and behavior as prospective variables that influence human behavior. Individuals’ ATTs significantly influence their decision intention and behavior. In other words, the more positive or negative an individual’s ATT, the more likely they are to perform actions that are consistent with that ATT. Additionally, Ajzen (1991) defines “subjective norms” as “the social pressure experienced after performing or not performing a behavior.” Han et al. (2010) further specifies that this pressure is primarily derived from “relatives, friends, colleagues,” and other individuals who are associated with the individual in society. In other words, individuals who are responsive to positive SN are more inclined to adopt positive decision-making intentions and behaviors. Finally, PBC refers to “the perceived ease with which a person can perform a behavior.” To develop an extended TPB model that is more relevant to the purchase behavior (PB) of green packaging products, we add two variables to the conventional TPB framework: EC and WPP.

### 2.2 Environmental concern

EC is the extent to which the public is aware of environmental issues and is willing to take action to address them (Kingston and Paulraj, 2023). It is a significant intrinsic motivation that drives green consumer behavior and is indicative of individuals’ awareness, ATTs, and values regarding environmental issues (George et al., 2023). Numerous studies have demonstrated that consumers who demonstrate greater EC are more inclined to engage in pro-environmental purchasing behaviors. For instance, groups that pay more attention to environmental issues are more inclined to purchase eco-friendly products, choose those with environmentally friendly packaging, and are willing to pay a premium for green products (Mishra et al., 2023).

Initially, consumers’ favorable ATTs toward green products substantially improved by addressing ECs. Environmentally conscious consumers are more likely to acknowledge the environmental value and social significance of green products, resulting in favorable assessments of green packaging products (Petkowicz et al., 2024). Research has demonstrated that EC is a significant antecedent in the prediction of consumers’ green ATTs and green purchase intentions (Kotyza et al., 2024). Second, consumers’ SN may improve through EC (Pinem et al., 2024). Consumers are more susceptible to the influence of SN and pay closer attention to their peers’ expectations of green consumption because of increased environmental awareness. Consequently, the propensity to purchase green packaging products has increased. Third, consumers’ PBC may be improved by ECs. When consumers are deeply concerned about environmental issues, they are more likely to take the initiative to overcome barriers to purchasing green-packaged products, such as searching for purchasing channels and comparing product information, thereby enhancing PBC.

Furthermore, EC and WPP are closely related. Environmentally conscious consumers are more likely to believe that green products have greater value and are willing to pay a premium for environmentally responsible green packaging products. The formation mechanism of consumer willingness to purchase green packaging products can be explained more comprehensively by incorporating ECs into the TPB extension model. EC is a significant internal driving force that not only directly influences consumers’ purchase intention but also indirectly influences it through various channels, including ATT, PBC, and SN (Petkowicz et al., 2024). It also has a significant impact on consumers’ WPP prices. Consequently, the theoretical significance and practical value of introducing the concept of EC into this study are sufficient.

Based on the theoretical analysis and literature review above, this study proposes the following hypotheses:

*H1*: EC has a positive influence on ATT.

*H2*: EC has a positive influence on SN.

*H3*: EC has a positive influence on BI.

*H4*: EC has a positive influence on PBC.

*H5*: EC has a positive influence on WPP.

### 2.3 Attitude

ATT is a critical construct for comprehending human behavior. It is defined as a comprehensive assessment of an individual’s approval or disapproval of a specific object or behavior (Ajzen and Fishbein, 1970). The correlation between ATTs and environment-related variables has been investigated extensively in green consumption research. The researchers believe that positive ATTs have the potential to influence behavior; however, BI is a critical mediator in the transformation of ATTs into actual PB (Ajzen, 2015). The more favorable consumers’ ATTs, the greater their intention to make environmentally friendly purchases (Hagger et al., 2022). Several empirical studies have demonstrated this. For instance, research conducted on consumers in the United States, India, and China has consistently demonstrated that positive ATTs are substantially and positively correlated with a higher willingness to pay and green purchase intention (Tang and Lam, 2017; Varshneya et al., 2017; Kirmani and Khan, 2018). Studies of various green consumption sectors, including organic products and green hotels, have yielded comparable results (Bian and Forsythe, 2012; Han and Yoon, 2015). Consequently, it can be reasonably concluded that consumers’ favorable ATTs toward green-packaged products significantly increase their readiness to acquire them.

Consumers’ subjective value judgments and overall affective tendencies toward green packaging are the primary focus of this study, which examines their ATTs toward green packaging products. Consequently, the following hypothesis is proposed:

*H6*: ATT has a substantially positive facilitating effect on BI, and it mediates the relationship between EC and BI.

### 2.4 Subjective norm

According to the TPB, SNs also play a significant role in determining BIs. An individual’s perceived social pressure from significant others, which may motivate them to perform or refrain from performing a specific behavior, is referred to as the SN (Ajzen, 1991). Close acquaintances, family members, colleagues, and other influential individuals may serve as such significant others (Li et al., 2023). SNs are the result of people’s motivation to comply with their normative beliefs regarding psychological mechanisms (Ajzen, 1991). The extent to which individuals are willing to comply with these expectations is represented by their motivation to comply, whereas normative beliefs reflect their perceptions of the importance of the actions that others expect them to take. Consequently, SNs reflect the behavioral limitations individuals recognize within their social contexts.

SNs are widely recognized as a principal factor affecting BIs in consumer behavior research. Concerning green consumption, consumers are more likely to engage in green purchasing behavior when they are aware that their significant others support them (Paul et al., 2016). According to several studies, SNs are substantially and positively correlated with consumers’ intentions to buy environmentally friendly products, according to several studies (Roh et al., 2022; Jebarajakirthy et al., 2024). For instance, one study found that consumers’ propensity to purchase organic food was positively influenced by SN (de Oliveira et al., 2025). This implies that consumers’ intentions to purchase environmentally friendly products are significantly influenced by their social identity and peer pressure.

More importantly, SNs may serve as mediators between ECs and BI. An individual’s perception of environmental issues and willingness to protect the environment are reflected in their environmental concern (Purwanto and Rini, 2022). Environmentally conscious consumers are more inclined to regard environmental protection as a significant social concern and are more inclined to adhere to SNs that align with their environmental values. Chen and Tung’s (2014) research demonstrated that the variables in the TPB, which include SN, mediate the relationship between ECs and behavioral intention. Their research demonstrated that consumers’ intentions to stay at green hotels are indirectly influenced by ECs, with SN serving as a significant mediating variable. Additionally, the centrality of SN in promoting sustainable consumption behavior is emphasized by Norm Activation Theory (Dursun et al., 2024). Consequently, SN may serve as a mediating mechanism for ECs to influence BIs, in addition to directly influencing them. Consequently, the following hypothesis is proposed:

*H7*: The SN had a substantially positive facilitating effect on BI and it mediated the relationship between EC and BI.

### 2.5 Perceived behavioral control

PBC is a fundamental component of the TPB, which denotes the perceived ease or difficulty of an individual in performing a specific behavior (Ajzen, 1991). PBC is essentially a subjective evaluation of an individual’s capacity to regulate behavior and considers both internal and external control factors (Ayalew and Zewdie, 2022). External factors may encompass resources and conditions, such as time, money, opportunities, and social support, whereas internal factors may include an individual’s self-confidence, knowledge, and talents (Liao et al., 2023). Therefore, PBC encompasses a thorough assessment of a behavior’s controllability and achievement, in addition to individual competence.

PBC is a pivotal factor in forecasting the likelihood of behavior engagement, as substantiated by numerous studies. Individuals are more likely to cultivate a tendency to engage in an action when they perceive it as straightforward and feasible (Hines et al., 1987). There is widespread validation of the positive impact of PBC on the propensity to purchase green products regarding green consumption. For example, research has demonstrated that consumers who exhibit higher levels of PBC in contexts such as recycling behaviors and organic food purchases are also more inclined to engage in such behavior (Batooli et al., 2022; Ifthiharfi et al., 2025). Particularly, consumer perceptions of PBC-related factors, including the affordability of product prices, convenience of purchasing channels, and difficulty in obtaining product information, directly influence their final purchasing decisions, particularly in the context of green product purchasing (Gao and Huang, 2025).

Additionally, EC, which is a significant intrinsic motivation that drives green consumer behavior, may also influence BIs by influencing PBC and, consequently, buying intentions. The likelihood of consumers actively seeking and creating accommodations to overcome barriers to purchasing green products increases as they become more environmentally conscious. For instance, environmentally conscious consumers may be more inclined to invest additional time and effort in the search for green-packaged products or consume slightly higher-priced green products because they are convinced that their actions can contribute to environmental protection. Hines et al. (1987) conducted a meta-analytic study that demonstrated that consumers who strongly believe in their ability to positively influence the environment (i.e., high PBC) are more likely to embrace sustainable consumption behaviors. The study conducted by Chen and Tung (2014) observed that PBC served as a mediator between ECs and the propensity to select green hotels. Consequently, we conclude that PBC may serve as a mediator between EC and consumers’ inclinations to acquire environmentally friendly packaged goods. Consumers with higher EC levels may be more inclined to believe that purchasing green packaging products is feasible and manageable, which increases their BI. The following hypotheses are proposed in this study based on TPB and the logic that EC may influence PBC and, consequently, BI.

*H8*: PBC has a substantially positive facilitating effect on BI and it mediates the relationship between EC and BI.

### 2.6 Willingness to pay premium

Price has consistently been a significant factor in the decision-making process of consumers (Yadav and Pathak, 2017). Comprehending consumer WPP for socially responsible products is essential for businesses. Consumer propensity to pay an additional premium for green products can be interpreted as an indicator of pro-environmental behavior (Ajzen, 1991), as the price is one of the primary obstacles to green consumption. The primary reason for the higher cost of green or environmentally friendly products is the increased investment in their production process. Consequently, the market acceptance and promotional potential of green products are directly correlated with consumers’ propensity to pay green premiums.

Young consumers are generally more price sensitive (Huang et al., 2024) and have a general perception that green products are more expensive than conventional products. High prices are frequently perceived as a barrier to green consumption (Sharma and Jayswal, 2025). A study conducted on a sample of Chinese consumers revealed that price sensitivity continues to be a significant factor in the direction of their green purchasing behavior (Cao and Wang, 2024).

Recent research has found that EC remains the most persistent driving force for WPP. A large sample of evidence from “Generation Z” shows that EC remains the most predictive indicator for consumers to pay more for green consumer goods (Gomes et al., 2023). Furthermore, when the environmental attributes are clearly marked on the packaging, the perceived green value and brand trust will further enhance consumers’ WPP (Singh and Pandey, 2018). Meanwhile, PWC’s 2024 Voice of Consumers survey indicates that consumers are willing to pay a 9.7% sustainability premium, even when the cost of living and inflation issues are severe. In conclusion, this study regards WPP as a downstream outcome of EC and also as a key mediator in transforming environmental concerns into specific behavioral intentions. The following hypotheses were proposed based on the aforementioned analysis:

*H9*: The WPP makes a substantially positive contribution to BI and mediates the relationship between EC and BI.

### 2.7 Behavioral intention and purchase behavior

In the consumer purchase decision-making process, BI is regarded as the most direct predictor of actual PB (Ajzen, 2002). The TPB model and other well-established theoretical models regard BI as an antecedent variable of behavior (Wang et al., 2024). The likelihood that the consumer will implement appropriate purchasing behavior increases as the strength of the BI increases (Ajzen, 2002). Many studies have demonstrated that BI has a positive predictive effect on a diverse range of consumer behaviors, including green purchasing behavior (Zhang et al., 2019). For example, consumer willingness to purchase green packaging products increases with positive ATTs toward green packaging products, subjective normative identity, and increased levels of PBC. This increased the probability of purchasing green packaging products. Consequently, the following hypothesis is proposed:

*H10*: BI has a positive influence on PB.

**Figure 1:**
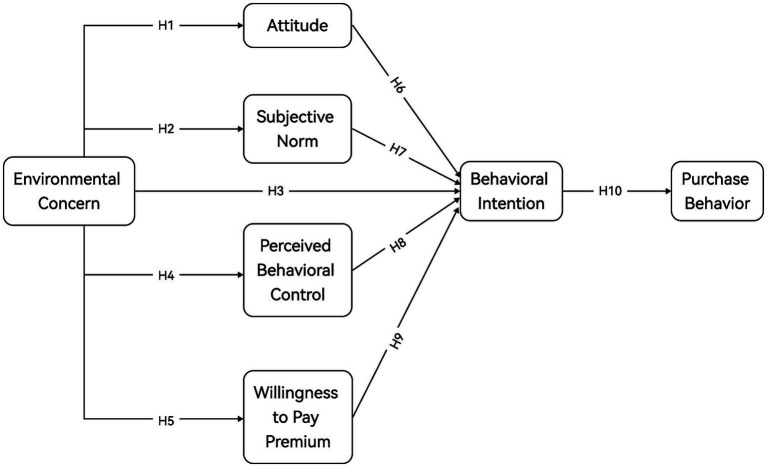
The TPB extended model’s theoretical framework diagram.

In sum, Figure 1 illustrates the theoretical framework of the extended TPB model utilized in this investigation.

## 3 Methods

### 3.1 Participants

The participants were from China’s GBA. This area encompasses the Hong Kong Special Administrative Region, Macao Special Administrative Region, and the cities of Guangzhou, Shenzhen, Zhuhai, Foshan, Huizhou, Dongguan, Zhongshan, Jiangmen, and Zhaoqing in Guangdong Province. A total of 398 data points were collected through online questionnaires, out of which 370 were valid resulting in an effective recovery rate of 92.96%. A sensitivity analysis was conducted in G*Power 3.1.9.7 (test family: F tests; statistical test: linear multiple regression—fixed model, *R*^2^ deviation from zero). Using *α* = 0.05, power (1–*β*) = 0.80, total sample size *N* = 370, and the largest number of predictors in the structural model (*k* = 5), the minimum detectable effect was *f*^2^ = 0.035 (equivalently *R*^2^ = 3.40%) (Prajapati et al., 2010). The corresponding critical value was *F* (5, 364) = 2.24 with noncentrally parameter *λ* = 13.02. Because the observed *R*^2^ for the endogenous variables in the SEM exceeds 3.40% (*R*^2^ = 85.9%), the sample size is adequate to test the hypothesized effects. Table 1 illustrates the distribution of the study sample about the demographic factors. Upon completing the questionnaire, all participants were awarded a nominal sum of cash as a token of appreciation for their time and work. This research underwent ethical assessment for human testing by Guangdong University of Technology (review number GDUTXS20250126), and all experimental procedures adhered to applicable rules and regulations.

**Table 1:** Description of sample structure characteristics.

Variable	Type	Frequency	Proportion (%)	M	SD
Gender	Male	142	38.40%	1.620	0.487
	Female	228	61.60%		
Age	<18	33	8.90%	3.110	1.529
	18–25	150	40.50%		
	26–30	57	15.40%		
	31–40	37	10.00%		
	41–50	62	16.80%		
	51–60	31	8.40%		
Employment	Full-time work	216	58.40%	1.850	1.053
	Part-time work	6	1.60%		
	Students	142	38.40%		
	Homemaker	3	0.80%		
	Self-employed	3	0.80%		
Education	High school/Secondary school	8	2.16%	4.280	0.751
	Junior college	37	10.00%		
	Normal courses	167	45.14%		
	Post-graduate or higher	158	42.70%		
Monthly income (CNY)	<1,000	59	15.95%	3.720	1.772
	1,000–2,500	43	11.62%		
	2,500–5,000	73	19.73%		
	5,000–7,500	53	14.32%		
	7,500–10,000	52	14.05%		
	>10,000	90	24.32%		

### 3.2 Measurement tools

We utilized questionnaires from previous studies to collect the data. The questionnaire is divided into two sections. The first section pertains to the socioeconomic characteristics of the respondents, including gender, age, employment status, education, and personal monthly income. The second section consisted of a five-point Likert scale adapted from previous studies on latent variable scales to assess respondents’ willingness to purchase green packaging products (Mancha and Yoder, 2015; Paul et al., 2016; Prakash and Pathak, 2017; Yadav and Pathak, 2017; Akhtar et al., 2021; Kabir and Islam, 2022; Nekmahmud et al., 2022; Qin and Song, 2022), including the following seven constructs: ATT, EC, SN, PBC, WPP, BI, and PB. A trap question was simultaneously included in the second section of the questionnaire to facilitate the future screening of valid items. For specific question item design, please refer to Appendix A.

### 3.3 Statistical analysis

Considering that this study aims to extend the TPB, we employed covariance-based structural equation modeling (CB-SEM) for estimation. Existing methodological literature widely acknowledges that CB-SEM is more suitable for theory-driven, model fit-oriented research contexts. It allows evaluation of the overall fit of both the measurement and structural models through multiple fit indices. In contrast, partial least squares structural equation modeling is more suited for predictive and exploratory purposes, typically offering greater advantages when sample sizes are limited or theoretical frameworks are still developing (Hair et al., 2011). Given sufficient sample size and high-quality measurement models, CB-SEM offers relative advantages in terms of unbiased parameter estimation and rigorous model testing (Kline, 2023). Therefore, this study employs CB-SEM to more robustly validate the extended TPB model and its path structure. We used SPSS 26.0 and AMOS 28.0 to assess the reliability, discriminant validity, and fit of the confirmatory factor analysis model; examine path associations; and test mediation effects.

## 4 Results

### 4.1 Analysis of reliability and validity

The analysis revealed that Cronbach’s *α* for all constructs exceeded the threshold of 0.7 for the base study, as presented in Table 2 (Kline, 2023). This indicates that the scale employed in this study demonstrated strong internal consistency and reliability. In the validity assessment, the average variance extracted value for each dimension exceeded 0.5, whereas the composite reliability value surpassed 0.7. This suggests that all the dimensions demonstrated strong convergent validity and combined reliability.

**Table 2:** Reliability and validity testing.

Variable	Item	rho_α	Loadings	CR	AVE
ATT	ATT1	0.904	0.741	0.906	0.659
	ATT2		0.840		
	ATT3		0.822		
	ATT4		0.871		
	ATT5		0.778		
EC	EC1	0.817	0.718	0.826	0.613
	EC2		0.797		
	EC3		0.829		
SN	SN1	0.939	0.906	0.941	0.800
	SN2		0.947		
	SN3		0.940		
	SN4		0.774		
PBC	PBC1	0.758	0.698	0.765	0.521
	PBC2		0.771		
	PBC3		0.694		
WPP	WPP1	0.956	0.909	0.956	0.845
	WPP2		0.928		
	WPP3		0.913		
	WPP4		0.926		
BI	BI1	0.929	0.854	0.930	0.692
	BI2		0.872		
	BI3		0.857		
	BI4		0.884		
	BI5		0.659		
	BI6		0.844		
PB	PB1	0.882	0.859	0.886	0.661
	PB2		0.779		
	PB3		0.844		
	PB4		0.765		

ATT, attitude; EC, environmental concern; SN, subjective norm; PBC, perceived behavioral control; WPP, willingness to pay premium; BI, behavioral intention; PB, purchase behavior.

### 4.2 Differentiation of validity

Furthermore, to evaluate the discriminant validity, each construct’s discriminant validity was examined utilizing the heterotrait–monotrait (HTMT) ratio of correlations, with the expectation that the HTMT value remains below 0.9 (Ali et al., 2018). Our research findings indicate that the HTML technique can effectively establish discriminative validity, as all values are below 0.9, signifying adequate discriminative validity.

### 4.3 Confirmatory factor analysis model fit test

The results of the model fitness test indicate that the chi-square degrees of freedom ratio was 2.786, which fell within the acceptable range of 1–3 (Kline, 2023). Additionally, the root mean square error was 0.070, which was considered to be within the favorable threshold of <0.08 (Bollen and Long, 1993). Additional tests of the IFL, TLI, and CFI yielded results of 0.933, 0.925, and 0.932, respectively, all of which exceeded the excellent threshold of 0.9 (Bentler and Bonett, 1980). Therefore, the combined results suggest that the model is a strong fit for this purpose.

### 4.4 Assessing the model’s explanatory ability

The coefficient of determination (*R*^2^) and the cross-validated redundancy index (*Q*^2^) are acknowledged as the primary metrics for assessing a structural equation model’s explanatory and predictive efficacy (Hair et al., 2019). An *R*^2^ value indicates the extent of variance in endogenous constructs accounted for by external factors, with thresholds of 0.19–0.33, 0.33–0.67, and above 0.67 denoting weak, moderate, and strong explanatory power, respectively. Similarly, a *Q*^2^ value greater than zero demonstrates that the model exhibits predictive relevance for the corresponding endogenous construct (Fornell and Larcker, 1981). The *R*^2^ values of Behavioral Intention (0.859) and Purchase Behavior (0.912) indicate strong explanatory power. Meanwhile, the *Q*^2^ values of Behavioral Intention (0.544) and Purchase Behavior (0.547) are all greater than zero, confirming the model’s predictive relevance. These data collectively demonstrate that the structural model possesses considerable explanatory power and robust prediction validity about the major endogenous variables.

### 4.5 Tests of structural equation modeling

**Figure 2:**
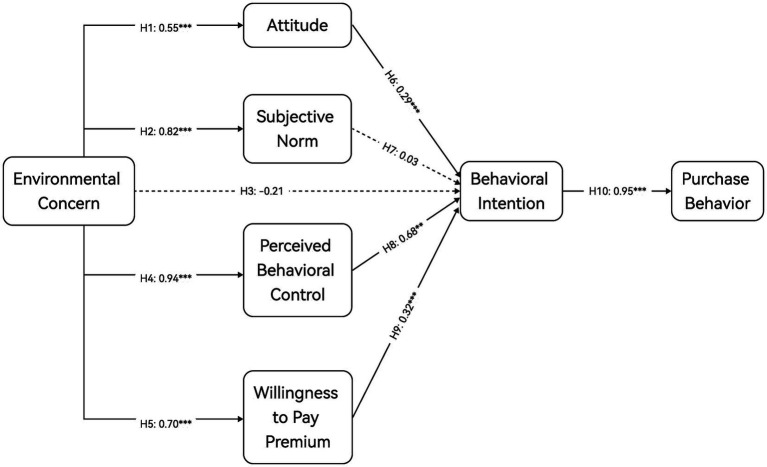
The standardized path diagram for the TPB extension model, ***p < 0.001. Dash line means not significant.

The analysis results presented in Table 3 indicate that the path hypothesis relationship test reveals significant positive influences of EC on ATT (*β* = 0.545, *p* < 0.001), SN (*β* = 0.820, *p* < 0.001), PBC (*β* = 0.939, *p* < 0.001), and WPP (*β* = 0.702, *p* < 0.001), supporting H1, H2, H4 and H5. Furthermore, ATT (*β* = 0.293, *p* < 0.001), PBC (*β* = 0.680, *p* < 0.05), and WPP (*β* = 0.318, *p* < 0.001) have a significant positive effect on BI, and BI, in turn, significantly influences PB (*β* = 0.955, *p* < 0.001), supporting H6, H8, H9 and H10. Nonetheless, EC (*β* = −0.212, *p* > 0.05) and SN (*β* = 0.027, *p* > 0.05) did not exhibit a significantly predictive relationship with BI, so H3 and H7 were not supported. The standardized path diagram for the TPB extension model is shown in Figure 2.

**Table 3:** Testing path relationships in structural equation modeling.

Hypotheses	Path	The point estimate	S.E.	*T*-value	Result
H1	EC → ATT	0.545	0.050	8.695***	Supported
H2	EC → SN	0.820	0.090	14.048***	Supported
H3	EC → BI	−0.212	0.303	−0.727	Not Supported
H4	EC → PBC	0.939	0.064	11.965***	Supported
H5	EC → WPP	0.702	0.088	12.305***	Supported
H6	ATT → BI	0.293	0.051	7.485***	Supported
H7	SN → BI	0.027	0.043	0.420	Not Supported
H8	PBC → BI	0.680	0.333	2.617*	Supported
H9	WPP → BI	0.318	0.031	6.957***	Supported
H10	BI→PB	0.955	0.051	19.942***	Supported

*p < 0.05, ***p < 0.001.

To assess mediation, we employed a bias-corrected and accelerated (BCa) bootstrap, treating an indirect effect as significant when its 95% confidence interval excluded zero. EC exhibited significant indirect effects on BI via three single-mediator paths—EC → ATT → BI (*T* = 4.150, *β* = 0.166, *p* < 0.001, 95% CI = [0.101–0.279]), EC → PBC → BI (*T* = 0.754, *β* = 0.665, *p* < 0.01, 95% CI = [0.212–3.900]), and EC → WPP → BI (*T* = 4.568, *β* = 0.233, *p* < 0.001, 95% CI = [0.149–0.357])—whereas the path via SN was not significant (*T* = 0.348, *β* = 0.023, *p* > 0.05, 95% CI = [−0.13–0.134]). When BI mediated effects on PB, the indirect effects of ATT (*T* = 5.329, *β* = 0.389, *p* < 0.01, 95% CI = [0.245–0.532]), PBC (*T* = 0.828, *β* = 0.879, *p* < 0.01, 95% CI = [0.277–4.271]), and WPP (*T* = 5.400, *β* = 0.216, *p* < 0.01, 95% CI = [0.142–0.303]) were significant, while those of SN (*T* = 0.346, *β* = 0.018, *p* > 0.05, 95% CI = [−0.097–0.108]) and EC (*T* = −0.253, *β* = −0.222, *p* > 0.05, 95% CI = [−3.317–0.29]) were not. Overall, ATT, PBC, and WPP function as robust mediators in the model, with the PBC pathway showing the largest effect; the SN-based pathway is unsupported.

### 4.6 Comparison of baseline model and extended model

Incremental validity was evaluated via nested model comparisons. Given the sensitivity of *χ*^2^ to sample size, we primarily relied on changes in incremental fit indices. Using Cheung and Rensvold’s ΔCFI ≤ 0.01 guideline and Chen’s ΔRMSEA ≤ 0.015 criterion, the extended specification showed a meaningful improvement over the baseline (ΔCFI = 0.032; RMSEA: 0.096 → 0.070) (Cheung and Rensvold, 2002). Information criteria further indicated a trade-off: AIC / BIC increased, consistent with the penalty for additional parameters (Akaike, 1974). Finally, increases in *R*^2^ translated into very large (BI, *f*^2^ ≈ 1.06) and medium–large (PB, *f*^2^ ≈ 0.48) block effects, surpassing Cohen’s benchmarks for a ‘large’ effect.”(Selya et al., 2012) The comparison results of the baseline (TPB) model and the extended model in terms of model fitting and explanatory power are shown in Table 4.

**Table 4:** Comparison of baseline (TPB) model and extended model in terms of model fit and explanatory power.

Indicators	Baseline (TPB)	Extended	Difference
χ^2^/df	904.354/205	1022.476/367	Δ*χ*^2^ = 118.122/Δdf = 162
CMIN/DF	4.411	2.786	Improved
RMSEA	0.096	0.070	Improved
CFI/TLI/IFI	0.900/0.887/0.900	0.932/0.925/0.933	Improved
AIC/BIC	1000.354/1188.202	1158.476/1424.594	Unimproved
*R*^2^ (BI)	0.710	0.859	Δ = 0.149/*f*^2^ = 1.060
*R*^2^ (PB)	0.870	0.912	Δ = 0.042/*f*^2^ = 0.480

## 5 Discussion

### 5.1 Interpretation of the results

Using the extended Theory of Planned Behavior as its framework, this study sets environmental concern in an upstream driving position and introduces willingness to pay a premium as a psychological variable that is relatively close to purchase decisions. These factors, along with attitude, subjective norm, and perceived behavioral control, jointly predict behavioral intention, which in turn predicts actual purchasing behavior. At the measurement model level, the reliability and validity indices all meet the required standards. The internal consistency and convergent validity of each construct satisfy common thresholds, and the HTMT values are generally below the empirical upper limit, indicating that the discriminant validity among the latent variables is acceptable. The structural model’s fit indices fall within the acceptable to good range, providing model-level support for the subsequent path analysis.

For the core pathways, the regression coefficients of EC on ATT, SN, PBC, and WPP are all positive and significant. This result suggests that higher environmental concern can significantly enhance individuals’ positive attitudes toward green or environmentally related targets, increase their perceived behavioral control (i.e., their perceived ability to carry out the behavior), and reinforce their willingness to pay a premium for environmental attributes. The beneficial impact of environmental concern on social norms is noteworthy, suggesting that customers who are environmentally conscious are more inclined to recognize societal or peer pressure. However, the intermediate paths leading to BI show clear differentiation: ATT, PBC, and WPP each have a significantly positive effect on BI, whereas the direct effects of SN and EC on BI are not significant. This result reveals that in the context of this study, the key psychological mechanisms forming purchase intention rely more on intrinsic individual factors (such as attitude, perceived behavioral control, and willingness to pay) than on external normative social constraints. Concurrently, the influence of EC on BI is transmitted primarily via indirect mediating pathways, rather than through a direct effect.

Among the three significant paths to BI, the effect of PBC is the strongest, followed by those of WPP and ATT. This ordering has significant theoretical implications: on the one hand, an individual’s sense of “can I do it?” (perceived behavioral control) is the most critical lever for converting environmental concern into concrete behavioral intention; on the other hand, economic considerations make an independent contribution at the intention level. The significance of WPP indicates that when consumers are willing to pay a premium for environmental attributes, the increase in behavioral intention is driven by the combined effect of the individual’s economic affordability and environmental identification.

Notably, subjective norm did not significantly predict consumers’ behavioral intention to purchase green packaging. This finding appears to deviate from traditional TPB applications, though it is not entirely an isolated case. Meta-analyses and reviews grounded in the TPB indicate that across numerous application contexts, the predictive effect of subjective norm on behavioral intention is generally weaker than that of attitude and perceived behavioral control, and sometimes even insignificant (Armitage and Conner, 2001; McEachan et al., 2016). Within the context of this study, this phenomenon may be further amplified by the sample’s structural characteristics. On the one hand, the sample includes a larger proportion of young adults and working professionals with higher educational attainment and relatively higher income levels. These groups tend to base their green consumption decisions more on personal beliefs, perceived efficacy, and economic trade-offs, while being less influenced by others’ expectations (Vrselja et al., 2024; Zhong et al., 2024). On the other hand, the Greater Bay Area operates within a highly marketized and digitized environment where consumers encounter fragmented subjective normative pressures. Online and offline channels, social circles, and media discourse often provide mixed or even conflicting guidance, potentially undermining the stable and singularly sourced subjective normative effects assumed by traditional TPB. Thus, the failure of subjective norms in this study not only aligns with the weaker subjective norm effects observed in TPB literature but also indicates that the form and sources of subjective norms may have evolved within the realm of green consumption.

In terms of the conversion from behavioral intention to purchase behavior, the path coefficient was significant and substantial in magnitude. Coupled with high *R*^2^ values (0.859 for BI; 0.912 for PB), this result indicates that the model has strong explanatory power for the key endogenous variables. Meanwhile, the *Q*^2^ values obtained through blindfolding (0.544 for BI; 0.547 for PB) were all greater than 0, demonstrating substantial predictive relevance for the endogenous variables. In other words, the model not only explains variance in observed outcomes but also predicts data points that have not yet been observed. These results collectively affirm the model’s efficacy in both explanatory and predictive aspects.

Mediation effect tests further refine the above conclusions. When treating BI as the outcome variable and EC as the predictor, the indirect effects of EC on BI through ATT, PBC, and WPP are all significant, whereas the indirect effect through SN is not significant. Likewise, with PB as the outcome variable and BI as the mediator, the indirect pathways from ATT, PBC, and WPP via BI to PB are significant, whereas those from SN and EC are not significant. This set of results indicates that the influence of EC is mainly transmitted to BI via three psychological pathways (ATT, PBC, and WPP), and is further channeled to PB through BI. Furthermore, SN, a subjective norm cue plays a relatively weak role in both explanation and mediation within this study. Finally, the PBC pathway is the most crucial in the consumer “motivation–intention–behavior” transmission process, suggesting that the self-perception of “I can do it” drives readiness for action and subsequent behavior more effectively than the social expectation of “I should do it.”

### 5.2 Implications for research

We expanded upon the TPB by integrating EC and WPP as supplementary dimensions. Model comparisons revealed a substantial increase in explanatory power: *R*^2^(BI) increased from 0.710 to 0.859 and *R*^2^(PB) from 0.870 to 0.912, indicating that the supplementary pathways significantly improved the prediction of intention and behavior. These findings not only affirm the applicability of the TPB to sustainable consumerism but also demonstrate that incorporating EC and WPP enriches the comprehension of critical psychological processes.

From a theoretical perspective, the findings contribute to the TPB research framework. First, they emphasize the pivotal role of EC in green consumption. Second, the addition of WPP shows that economic factors are not just outside forces that limit behavior, but also internalized motivations (Han et al., 2010). This study illustrates the applicability of the TPB across several disciplines and offers empirical evidence for the incorporation of supplementary value-oriented and economic factors into behavioral models. The enlarged model bolsters the theoretical framework of green purchasing behavior and provides a valuable template for the further integration of the TPB into environmental psychology and consumer research.

### 5.3 Implications for practice

Based on the extended TPB model and its empirical validation, this study provides several practical implications. First, environmental concerns play a significant role in shaping consumer attitudes and intentions. Therefore, businesses and governments should place greater emphasis on communicating environmental values in product promotions. For example, companies can strengthen consumers’ emotional identification and sense of responsibility by highlighting sustainability elements in marketing or through explicit brand commitments (Correia et al., 2023). Governments can also foster a social atmosphere aligned with green values through environmental education and policy advocacy, thereby enhancing public acceptance of green packaging (Shen and Wang, 2022). Second, given the significant role of perceived behavioral control in shaping consumer behavior, businesses should prioritize user-friendly packaging design during product development. Incorporating emotional design—targeting instinctive, behavioral, and reflective layers—can enhance consumers’ brand loyalty and cultural identification. Research indicates that visual design elements in packaging (such as color, graphics, layout, and brand logos) significantly increase purchase intention by enriching consumers’ brand experiences (Liu et al., 2025). Third, consumers’ willingness to pay a premium is crucial for translating behavioral intention into actual purchasing behavior. Companies can address this from two angles. On the one hand, they can reduce product prices through large-scale production and optimized supply chain structures, thereby lowering consumers’ price sensitivity toward green products. On the other hand, companies can offer differentiated product portfolios featuring basic green versions as well as premium deep-green versions. This allows price-sensitive consumers to access more affordable green options, while consumers with stronger environmental awareness and a higher willingness to pay can choose products with enhanced ecological attributes. Governments can also implement consumption policies—such as national green consumption subsidies—which not only stimulate consumption and support economic circulation domestically and internationally but also accelerate the market’s transition toward greener practices.

### 5.4 Limitations and future research

Although current research has yielded meaningful findings, several limitations remain. First, the study sample was drawn primarily from specific regions and was concentrated among higher-educated, higher-income groups, such as students, recent graduates, and full-time employees. These groups typically exhibit greater environmental awareness and are more willing to bear the additional costs associated with green products. Although the representativeness of student samples is often questioned, existing research indicates that, for many psychological and behavioral variables—particularly attitudes, values, and decision-making mechanisms—student samples do not differ significantly from broader populations (Druckman and Kam, 2009). Therefore, experimental designs based on student or higher-education convenience samples remain reasonable and widely accepted (Ashraf and Merunka, 2017). Nonetheless, the sample structure in this study may slightly overestimate consumer acceptance of green packaging. Thus, future research should place greater emphasis on diversifying participants’ educational backgrounds, income levels, and occupations, particularly by including individuals from low- and middle-income groups, to test the model’s applicability across different economic segments.

Cross-sectional studies demonstrate robust stability when examining complex latent variable structures and multivariate path relationships (Kwok et al., 2018). However, causal inference based on cross-sectional data has limitations. Although structural equation models can elucidate path relationships, it cannot fully establish dynamic causal mechanisms (Savitz and Wellenius, 2023). By incorporating temporal factors, future research can more comprehensively capture how consumers’ psychological processes evolve over time, thereby promoting the long-term stability and development of green consumption behaviors.

The role of the subjective norm pathway in this study was relatively weak, suggesting that future research could benefit from more nuanced distinctions regarding the form and source of subjective norms. On the one hand, subjective norms may be moderated by age and generational differences; for example, middle-aged and older individuals often rely more on the opinions of relatives, friends, and authoritative sources when making consumption decisions (Carpenter and Yoon, 2011). On the other hand, the present sample—primarily composed of young adults and middle-aged individuals—may have restricted variability in normative differences. Future studies could therefore employ multi-group analysis or stratified sampling to compare the strength of subjective norm effects across different age groups, socioeconomic statuses, and cultural backgrounds, as well as examine how subjective norms interact with attitudes and perceived behavioral control.

## 6 Conclusion

Using the Theory of Planned Behavior as a foundation, this research suggested and validated an extended framework that is more closely connected with the decision-making process of environmentally conscious consumers. According to this model, environmental concerns act as the upstream driver, and it is connected to attitude, perceived behavioral control, and willingness to pay a premium. In the end, behavioral intention is what leads to real purchasing behavior. The “values–attitudes–intentions–behavior” chain mechanism is represented by this framework in a more thorough manner than it is by the standard model. It fills the explanatory gap that may exist when relying solely on attitude, subjective norm, and perceived behavioral control.

In general, when it comes to sustainable consumption, there is no one psychological aspect that can fully capture the decision-making process during the purchasing process. The integration of environmental concerns and the willingness to pay a premium into a cohesive theoretical framework is something that needs to be carried out. In the future, we might conduct independent replications across a variety of product categories and cultural contexts, as well as employ longer time spans and data from other sources, to better characterize the framework’s boundary conditions and external validity. These kinds of initiatives will be helpful in translating the findings of this study into theoretical support that can be transferred and analytical standards that can be put into practice.

## Data Availability

The original contributions presented in the study are included in the article/supplementary material, further inquiries can be directed to the corresponding author.

## Appendix A

**Table:**

Construct	Item	Items
Attitude (ATT)	ATT1	I am willing to buy products with reusable packaging.
	ATT2	In my opinion, buying products with green packaging is a wise choice.
	ATT3	I think buying products with green packaging is good for the environment.
	ATT4	I am in favor of buying products with green packaging.
	ATT5	I am willing to buy recyclable products.
Environmental concern (EC)	EC1	I have replaced products for ecological reasons.
	EC2	Normally, I would buy a product that is less damaging to the environment.
	EC3	It’s important to me that the products I use do not pollute the environment.
Subjective norm (SN)	SN1	Most people who are important to me think I should buy products with green packaging when I shop.
	SN2	Most people who are important to me would want me to buy products with green packaging when I shop.
	SN3	Most of the people who are important to me prefer that I buy products with green packaging.
	SN4	Most people who are important to me think I should protect the environment.
Perceived behavioral control (PBC)	PBC1	I believe I can afford to buy green packaged products.
	PBC2	Green packaged products are usually found in the stores where I usually shop.
	PBC3	If I want to, I can buy green packaged products instead of traditional non-green packaged products.
Willingness to pay premium (WPP)	WPP1	I’m willing to pay a little extra for products that use green packaging.
	WPP2	For me, it is acceptable to spend extra money on products that use green packaging.
	WPP3	I’m willing to spend extra money on products that are less harmful to the environment.
	WPP4	I am willing to spend extra money on products with green packaging.
Behavioral intention (BI)	BI1	I will be buying products with green packaging in the near future.
	BI2	I plan to buy products in green packaging because they are more environmentally friendly.
	BI3	I will make an effort to buy green packaged products.
	BI4	I am more likely to make multiple purchases of products that use green packaging.
	BI5	If the price is right, I prefer to buy products that use green packaging.
	BI6	I am partial to buying products with green packaging.
Purchase behavior (PB)	PB1	I have green purchasing behavior for products I need on a daily basis.
	PB2	I have had green purchases within the last 6 months.
	PB3	I tend to buy products that use green packaging.
	PB4	I always choose products with reusable packaging.
